# Characterization of serum biomarkers and antibody responses against *Prevotella* spp. in preclinical and new-onset phase of rheumatic diseases

**DOI:** 10.3389/fcimb.2022.1096211

**Published:** 2023-01-18

**Authors:** Lena Amend, Benoît Thomas P. Gilbert, Penelope Pelczar, Marius Böttcher, Samuel Huber, Torsten Witte, Axel Finckh, Till Strowig

**Affiliations:** ^1^ Department of Microbial Immune Regulation, Helmholtz Centre for Infection Research, Braunschweig, Germany; ^2^ Division of Rheumatology, Geneva University Hospitals, Geneva, Switzerland; ^3^ I. Department of Medicine, University Medical Center Hamburg-Eppendorf, Hamburg, Germany; ^4^ Department of Rheumatology and Immunology, Hannover Medical School, Hannover, Germany

**Keywords:** gut microbiota, *Prevotella copri*, spondyloarthritis, rheumatoid arthritis, preclinical disease stages, biomarkers

## Abstract

**Introduction:**

The characterization of the influence of the microbiota on the development and drug responses during rheumatic diseases has intensified in recent years. The role of specific bacteria during disease development has become a central research question. Notably, several lines of evidence point to distinct microbes, e.g., *Prevotella copri (P. copri)* being targeted by antibodies in clinical phases of rheumatic diseases.

**Methods:**

In the present study, we compiled a broad collection of human serum samples from individuals at risk of developing RA, chronic RA patients as well as patients with new-onset of rheumatic diseases. We evaluated the presence of inflammatory biomarkers in our serum collection as well as serum antibody responses against novel, genetically distinct isolates of *P. copri* and several oral pathobionts.

**Results:**

Our analysis revealed the presence of increased levels of inflammatory markers already in pre-clinical and new onset rheumatoid arthritis. However, antibody reactivity against the microbes did not differ between patient groups. Yet, we observed high variability between the different *P. copri* strains. We found total serum IgG levels to slightly correlate with IgG antibody responses against *P. copri*, but no relation between the latter and presence or prevalence of *P. copri* in the intestine.

**Discussion:**

In conclusion, our work underlined the importance of strain-level characterization and its consideration during further investigations of host-microbiota interactions and the development of microbiome-based therapeutic approaches for treating rheumatic diseases.

## 1 Introduction

Rheumatic diseases include various different disorders, with rheumatoid arthritis (RA) and spondyloarthritis (SpA) being the most common ones. SpA includes further heterogeneous subgroups with distinct clinical presentations and genetic risk factors, such as ankylosing spondylitis (AS), psoriatic arthritis (PsA), reactive arthritis (ReA), and axial spondyloarthritis (axSpA) (Carron et al., 2020). Rheumatic diseases usually develop progressively over several years, starting with a preclinical phase and loss of tolerance, followed by local and systemic inflammation, and resulting in clinical disease manifestation (Deane and El-Gabalawy, 2014). The identification of biomarkers enabling diagnosis and disease monitoring during these different stages of rheumatic disorders is crucial for disease control. Established biomarkers for detecting and distinguishing rheumatic disorders include autoantibodies, cytokines and acute phase proteins (Mohan and Assassi, 2015). Moreover, the antimicrobial protein calprotectin appears to be a promising candidate biomarker for systemic inflammation and disease activity in rheumatic diseases (Abildtrup et al., 2015; Jarlborg et al., 2020). Secreted by neutrophils, monocytes and epithelial cells in response to proinflammatory signals, it has been found to be upregulated in the serum of RA patients (García-Arias et al., 2013; Nielsen et al., 2018), in early RA patients (symptom duration < 2 years) (Jonsson et al., 2017), as well as in chronic PsA and axSpA patients (Hansson et al., 2014; Jarlborg et al., 2020). Whether calprotectin and other markers can be used to identify and discriminate rheumatic diseases from unrelated diseases with similar symptoms at the onset of disease or even in pre-clinical stages has not been comprehensively investigated.

Besides other factors, the microbiota at oral and intestinal mucosal sites has been suggested to play a role in the pathogenesis of rheumatic disorders, including RA and SpA (Vural et al., 2020). Focusing mostly on RA, several studies have identified various commensal bacteria residing in the intestinal mucosa as well as in the oral cavity to be enriched in patients suffering from RA, implying these microbes as contributors to disease pathology. This concept has been supported by the link between the occurrence of periodontitis and the development of RA further implying periodontal pathogens in RA pathogenesis (Bingham and Moni, 2013). Sequencing of salivary samples of rheumatic patients identified specific bacterial signatures in the oral cavity of RA patients in different disease stages with several authors reporting the enrichment of oral *Prevotella* species in individuals at risk for RA (with systemic autoimmunity, but absence of clinical arthritis) (Tong et al., 2020), early RA patients (<12 months symptoms) (Esberg et al., 2021), patients with new onset RA and chronic RA (Scher et al., 2012; Tong et al., 2020). Additionally, the involvement of intestinal *Prevotella* species has been intensively explored by multiple studies investigating the gut-joint axis. Using 16S rRNA amplicon as well as metagenomic sequencing based approaches of patient fecal samples, intestinal *Prevotella* species have been found to be enriched in individuals genetically at risk for developing RA (Wells et al., 2020), in pre-clinical phases of RA (Alpizar-Rodriguez et al., 2019) as well as in chronic RA patients (Kishikawa et al., 2020). Particularly, increased relative abundances of *Prevotella copri* have been observed in fecal samples of new onset RA patients (disease duration of > 6 weeks and < 6 months) (Scher et al., 2013) and early RA patients (disease duration < 2 years) (Maeda et al., 2016). Although these results reinforced the specific connection between *Prevotella* and RA, a different study also observed several *Prevotella* species including *P. copri* to be enriched in AS patients (a subform of SpA) (Wen et al., 2017). Thus, the genus *Prevotella*, and especially the species *P. copri*, appear to be implicated in the occurrence of different rheumatic disorders. However, it needs to be noted that the species *P. copri* has recently been identified to be a species complex consisting of several subspecies (called clades) (Tett et al., 2019). Since these clades vastly differ based on their genomic content and their phenotype, a heterogeneous disease involvement is highly plausible.

Hitherto, it remains unclear whether specific microbial signatures are causes or consequences of arthritis development and whether they are linked to other confounding factors. Moreover, how these bacteria would mechanistically shape disease progression is not understood either. With the aim to investigate how specific microbes could promote rheumatic diseases, several studies have investigated the recognition of specific intestinal and oral bacteria by the immune system in RA patients. Pianta et al. identified a higher serum IgG and IgA response against *P. copri* in a subset of RA and NORA patients (Pianta et al., 2017). Concentrating on periodontal bacteria, Ogrendik et al. found *Porphyromonas gingivalis*, *Prevotella intermedia*, *Prevotella melaninogenica*, and *Bacteroides forsythus* (reclassified as *Tanerella forsythia*) to be specifically targeted by serum IgG in RA patients (Ogrendik et al., 2005).

We know from other diseases, such as rheumatic fever, that in certain circumstances the immune system can cross-react against homologous bacterial and self-structures (Faé et al., 2006). Thus, the observation that mucosal bacteria can be targeted by serum immunoglobulins in RA patients has raised the hypothesis of a cross reaction induced by these microbes, eventually promoting the progression of disease. Antimicrobial response factors have been found to be significantly enriched in the blood of RA patients compared to healthy controls, supporting an underlying systemic microbial exposure as stimulator of RA disease development (Ayyappan et al., 2020). Translocation of bacterial cells or microbial products from mucosal sites into the circulation is prevented by intact mucosal barriers, which separate the internal host environment from lumen content. Impairment of barrier function leads to an increased permeability, influx of luminal material and an activation of immune cells resulting in inflammation (Okumura and Takeda, 2018). As part of the mucosal origin hypothesis it has been suggested that the development of RA is triggered by imbalances or infections at mucosal sites, such as the intestine (Holers et al., 2018). Specifically, dysfunction of the intestinal barrier, also known as “leaky gut”, has been postulated to be a crucial factor in the pathology of RA, as zonulin, a marker for intestinal permeability, has been found to be upregulated in the serum of individuals with RA-specific autoimmunity but without clinical arthritis as well as chronic RA patients compared to controls (Tajik et al., 2020). Further gut permeability markers, in particular LPS, LBP and I-FABP, have been revealed to be increased in the serum of chronic RA patients, and partially in pre-RA (patients with seropositivity and joint pain) and/or early RA patients (with early, undifferentiated clinical arthritis) (Matei et al., 2021). Strikingly, restoring intestinal barrier integrity by specifically targeting zonulin partially prevented arthritis development (Tajik et al., 2020). Together, a complex interplay between impaired barrier functions and specific microbial signatures at mucosal sites, such as the presence of *Prevotella* species, are hypothesized to support or trigger RA (Brandl et al., 2021).

Considerations have to be given to the fact that the majority of the conducted studies generally focused on patients with established RA, and only a few looked into patient groups with new onset of the disease or individuals at risk for RA. More particularly, the classification criteria for distinct disease stages, such as pre-RA, early RA, NORA, and chronic RA, as well as respective sample sizes vastly differ between studies and thus impair the generalization of the results. Within this study, we therefore aimed to validate and expand previously obtained findings to different, clearly defined disease stages of RA as well as new onset of several other arthritic subforms. Therefore, we compiled a broad collection of human serum samples derived from different cohorts including individuals at risk of developing RA, patients with established RA disease, as well as patients with new onset of RA and the SpA subforms axSpA, PsA and ReA. We investigated the presence of calprotectin and zonulin as potential biomarkers for systemic inflammation and gut permeability, respectively, in the patients’ serum samples. Moreover, to gain insights into the interplay between the immune system and microbes in rheumatic disorders, we evaluated serum antibody reactivity against several pathobionts and different strains of *Prevotella copri* and looked into potential co-factors modulating this interaction.

## 2 Material and methods

### 2.1 Cohort descriptions

The SCREEN-RA study is an ongoing Swiss cohort, which enrolls and follows first-degree relatives (FDRs) of RA patients (detailed description in (Gilbert et al., 2021)). Hence, the participants of SCREEN-RA do not have a RA diagnosis at inclusion, but have increased genetical risk of developing the disease. The majority of them are healthy, while a fraction of the participants has autoimmunity associated with RA (meaning the presence of anti-citrullinated protein autoantibodies (ACPA) and/or rheumatoid factor (RF), and/or anti-ra33 antibodies in the serum), or clinically suspect symptoms, based on EULAR definition. In this study, we included serum samples from the following subgroups of the SCREEN-RA cohort: first-degree relatives without any autoantibodies or symptoms associated with RA (named FDR 1, serving as control group), first-degree relatives with presence of ACPA and/or RF in the serum (named FDR 2), and individuals with symptoms and signs associated with possible RA with/without presence of ACPA and/or RF (named FDR 3).

The Swiss Clinical Quality Management (SCQM) RA cohort is another Swiss registry, founded in 1997 with the support of Swiss regulatory authorities. SCQM is thus a prospective, longitudinal cohort of Swiss RA patients. The participants are enrolled by their practitioner rheumatologist after being diagnosed. At inclusion, RA patients provide a serum sample which is divided in multiple aliquots and stored in the SCQM biobank.

The Rheuma-VOR cohort is an ongoing German cohort, which recruits untreated patients with new onset of different rheumatic disorders. The cohort is dedicated to arthritis and to inflammatory back pain. Therefore, general practitioners could refer patients with either inflammatory back pain fulfilling the Berlin criteria or with symptoms of arthritis (swollen joints, morning stiffness > 30 min, elevated C-reactive protein (CRP) and/or erythrocyte sedimentation rate (ESR)) to rheumatologists. The rheumatologist diagnosed RA or other rheumatic disorders when the patient had been examined and all the laboratory values including RF, ACPA, CRP, ESR, and antinuclear antibodies (ANA) were available. The diagnosis of RA was based on the judgment of the rheumatologist. Stool samples as well as serum samples were obtained at the time of their first medical examination and various clinical data as well as dietary habits are recorded. Within this study, samples were included from newly diagnosed rheumatoid arthritis (RA), psoriatic arthritis (PsA), axial spondyloarthritis (axSpA), or reactive arthritis (ReA) patients. Patients diagnosed with non-rheumatic diseases (NRD) including arthralgia, lumbago, arthrosis, angioedema, tendopathy, and nonspecific backpain served as a control group. Serum samples from healthy blood donors (BD) were included as an additional control group for measuring serum biomarkers. Individuals were recruited *via* the German Hospital conducting the Rheuma-VOR recruitment. Additionally, only individuals who have not taken any medication in 7 days preceding the donation were permitted to donate blood. Due to obligatory anonymization of these blood donors, age and gender were the only collected parameters.

Serum samples from IBD patients were obtained from the University Medical Center Hamburg-Eppendorf in Germany. Individuals diagnosed with ulcerative colitis or Crohn’s disease were recruited within the scope of a human study approved by the local ethical committee. Ulcerative colitis patients with a Mayo Score ≥3 were considered as active disease patients, whereas the Bradshaw index was used to classify active Crohn’s disease (Bradshaw index ≥5) and Crohn’s disease patients in remission (Bradshaw index 0-4).

### 2.2 Isolation of *P. copri* strains

*P. copri* strains were isolated from fecal samples of *P. copri* positive donors previously identified by 16S rRNA gene sequencing. Fresh fecal samples were stored in sealed glass vials prefilled with 50% glycerol/BHI (Brain Heart Infusion) medium in -80°C. For isolation, the fecal sample was thawed and streaked out on BHI blood agar plates supplemented with vancomycin by performing dilution ranging from 10^0^ to 10^-3^. The plates were incubated at 37°C inside an anaerobic chamber for 48-72h. Single colonies were picked into BHI medium supplemented with fetal bovine serum (FBS) and Vitamin K3 (BHI-S) and were further incubated at 37°C until grown. The resulting cultures were screened by PCR using *P. copri* specific primers (P_copri_69F: CATCGAAAGCTTGCTTTTGATGGGC, P_copri_853R: CTTGGCCGCTGACCTGTTCAGA). After species confirmation by Sanger sequencing, the obtained cultures were aliquoted into sealed glass vials mixed with 50% glycerol/BHI medium and were immediately frozen in –80°C.

### 2.3 Bacterial strains

The *P. copri* type strain (DSM 18205), *Porphyromonas gingivalis* (DSM 20709), *Prevotella intermedia* (DSM 20706) and *Prevotella melaninogenica* (DSM 7089) were obtained from the Leibniz Institute DSMZ-German Collection of Microorganisms and Cell Cultures GmbH. All DSMZ strains as well as *P. copri* in-house strains were cultured and maintained in BHI-S medium inside an anaerobic chamber.

### 2.4 Preparation of bacterial cultures for ELISA

All strains were maintained inside an anaerobic chamber using BHI-S medium. Bacterial cultures were grown freshly from an overnight inoculation until exponentially phase and until reaching an OD600 ranging from 0.4 to 0.8. An aliquot of the culture was plated in serial dilutions (10^-4^, 10^-5^, 10^-6^) on BHI blood agar plates and served later on as quantification control. The rest of the culture was taken out of the chamber, was pelleted by centrifugation and was washed once with PBS. To inactivate the bacterial cells, the pellet was further resuspended in 1% Formalin and kept in the fridge. After 24h, Formalin was removed by centrifugation, the pellet was washed twice with PBS and the resulting solution was normalized to 4x10^6^ cells/ml by quantifying the colony forming units (CFU) on the previously prepared BHI blood agar plates. Bacterial solutions were kept at -20°C for long-term storage. The identity of all cultures was additionally checked by Sanger sequencing. Because *Prevotella intermedia* failed to be pelleted, we exceptionally used a fresh culture instead of a Formalin fixed culture for subsequent anti-bacterial ELISA assays.

### 2.5 Anti-bacterial IgG and IgA ELISA

Corning ELISA plates (Microplates, Cat: 3690) were coated with 25µl of the previously described bacterial solutions per well (resulting in a final concentration of 10^5^ bacterial cells/well) and were incubated overnight at 4°C. The next day, plates were washed twice with PBS solution supplemented with 0.05% Tween 20 (PBST) and were blocked with PBS + 5% milk powder for 1h at RT on a shaker. After washing the plates twice with PBST, 25µl of patient serum samples were added as duplicates to the plates (dilution 1:100 in PBS + 5% milk) and were incubated for 2h at RT on a shaker. Following washing four times with PBST, 25µl diluted HRP-conjugated anti-human IgG or IgA were added to the plate and were incubated for 1.5h at RT on a shaker (anti-human IgG used for oral pathobionts: Goat anti-human IgG antibody, GenScript, Cat. No.: A00166, dilution 1:5,000; anti-human IgG used for *P. copri* strains: Goat anti-human IgG, Invitrogen, Ref: SA5-10283, dilution 1:10,000; anti-human IgA: Goat anti-human IgA, BioLegend, Cat: 411002, dilution 1:1,000). After washing 3 times with PBST and twice with PBS, TMB mix was added to the wells (25µl/well) and was incubated for 10 min (for IgG) or 15 min (for IgA). After adding 25µl of H2SO4, the plates were measured at the ELISA reader at 450 nm against 570 nm as a reference. All samples were measured in duplicates and additional four control serum samples were included on each plate for inter-plate standardization.

### 2.6 ELISA for whole serum IgG, serum calprotectin & serum zonulin levels

Total serum IgG was measured using the IgG human uncoated ELISA Kit from Invitrogen, Cat: 88-50550, following the manufacturer’s instructions using a serum dilution of 1:100,000. Serum calprotectin was quantified using the RayBio Human S100-A8/A9 complex ELISA Kit from RayBiotech, Cat: ELH-S100A8-9, following the manufacturer’s instructions. Serum zonulin was measured using the IDK Zonulin ELISA Kit from Immundiagnostik AG, Cat: K5601, following the manufacturer’s instructions.

### 2.7 16S rRNA amplicon sequencing and analysis of *P. copri* abundance

The relative abundances of *P. copri* in the fecal samples from the SCREEN-RA cohort have been obtained from previously generated data by our group (Alpizar-Rodriguez et al., 2019). Using identical workflows and analyzing pipelines as described by Alpizar-Rodriguez et al., amplification and sequencing of the 16S rRNA gene as well as the analysis of *P. copri* relative abundance were performed from the fecal samples of Rheuma-VOR and SCQM cohort. Sequencing data of all samples used in this study was deposited to a public repository with the Accession no./Bioproject: PRJEB55184.

### 2.8 Phylogenetic analysis

The phylogenetic analysis was conducted using whole genome sequencing data from the *P. copri* strains used in this study and genome data from previously published *P. copri* strains (Tett et al., 2019) as a reference. Phylogenetic analysis was conducted using bacterial marker genes predicted and aligned by GTDBTk version 1.7.0. The tree was built using the neighbor-joining approach with 1000 boot steps in MEGA-X.

### 2.9 Statistical analysis

Statistical analysis was performed using GraphPad Prism (GraphPad Software, Inc.). If not further indicated, data are presented as mean ± SD (standard deviation) and statistical differences were analyzed using Kruskal-Wallis test. P values ≤ 0.05 were considered as significant: *p < 0.05, **p < 0.01, ***p < 0.001, ****p < 0.0001.

## 3 Results

### 3.1 Inflammatory biomarkers in the serum of different rheumatic patient groups during various disease stages

To study calprotectin levels in preclinical phases of RA, 165 serum samples from the Swiss SCREEN-RA and SCQM cohorts were characterized. Specifically, samples from the two cohorts were divided into four groups: i) first degree relatives of RA patients without any ACPA or RF positivity and disease symptoms as controls (FDR 1), ii) first-degree relatives with RA associated auto-immunity defined by serum ACPA and/or RF positivity (FDR 2), iii) first-degree relatives having RA specific symptoms with/without ACPA and/or RF autoantibodies (FDR 3), and iii) chronic RA patients (cRA) (see **Table 1** for details). To study the new onset phase, serum samples were obtained from 178 individuals recruited *via* the complementary Rheuma-VOR study being newly diagnosed with a rheumatic disorder, including rheumatoid arthritis (RA), psoriatic arthritis (PsA), axial spondyloarthritis (axSpA), or reactive arthritis (ReA). Moreover, within this cohort also patients with unspecific non-rheumatic diseases (NRD), such as arthralgia, lumbago, and back pain were recruited as the diagnosis occurred after recruitment. Finally, serum samples from healthy blood donors (BD) recruited *via* the same German hospital as the Rheuma-VOR patients were additionally included. Detailed information about the individuals’ clinical metadata are listed in **Table 1**.

**Table 1:** Serum samples included within this study.

Cohort	SCREEN-RA/SCQM				Rheuma-VOR						IBD		
Patient group	FDR 1	FDR 2	FDR 3	cRA	BD	NRD	RA	PsA	axSpA	ReA	UC A	CD A	CD R
**total numbers, n (female %)**	42 (76)^a^	40 (88)^a^	38 (90)^a^	45 (78)^a^	20 (56)^a^*	35 (57)^a^	33 (67)^a^	50 (50)^a^	30 (53)^a^	10 (80)^a^	(N/A)^a^	(N/A)^a^	(N/A)^a^
	(N/A)^b^	(N/A)^b^	(N/A)^b^	(N/A)^b^	20 (56)^b^*	22 (55)^b^	33 (67)^b^	48 (50)^b^	30 (53)^b^	10 (80)^b^	14 (43)^b^	13 (77)^b^	12 (50)^b^
**age, median**	54	54	59	56	60	46^a^ / 49^b^	54	50	34.5	51	36	33	44
**ACPA positivity, n**	0	25	9	31	(N/A)	0	22	0	1	0	(N/A)	(N/A)	(N/A)
**RF positivity, n**	0	19	8	34	(N/A)	1	24	1	1	0	(N/A)	(N/A)	(N/A)

*Metadata only available for 16 participants.

a Samples used in calprotectin assay.

b Samples used in zonulin assay.N/A stands for "not available".

**Figure 1:**
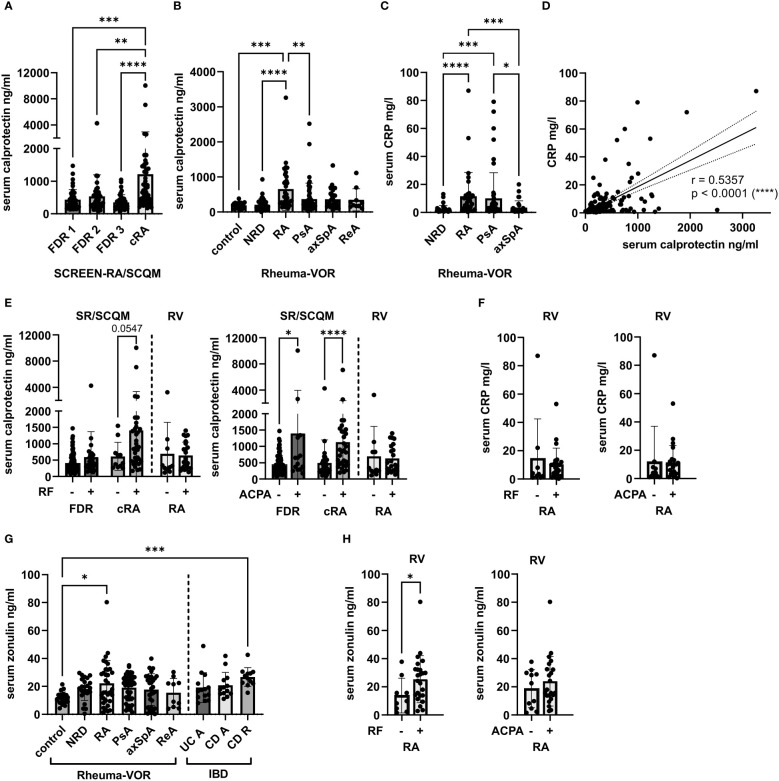
Calprotectin and zonulin levels in the serum of individuals at risk of RA, new onset rheumatic patients and chronic RA patients. **(A)** Serum calprotectin levels in seronegative, symptom-free first-degree relatives (FDR 1, n=42), individuals with RA specific autoantibodies (FDR 2, n=40), individuals with RA specific symptoms (FDR 3, n=38) and chronic RA patients (cRA, n=45), recruited *via* the SCREEN-RA and SCQM cohort. **(B)** Serum calprotectin levels in healthy blood donors (BD, n=20), and patients newly diagnosed with non-rheumatic disease (NRD, n=35), rheumatoid arthritis (RA, n=33), psoriatic arthritis (PsA, n=50), axial spondyloarthritis (axSpA, n=30) and reactive arthritis (ReA, n=10), recruited *via* the Rheuma-VOR cohort. **(C)** Serum C-reactive protein (CRP) levels in NRD (n=34), RA (n=33), PsA (n=50) and axSpA (n=28) patients from the Rheuma-VOR cohort. **(D)** Spearman correlation between serum CRP and serum calprotectin measured in patients from the Rheuma-VOR cohort. **(E)** Serum calprotectin levels in FDR individuals and cRA patients from the SCREEN-RA/SCQM cohort and RA patients from the Rheuma-VOR cohort divided by RF and ACPA positivity. Statistical differences between positive and negative groups within patient groups were calculated using Mann-Whitney test. **(F)** Serum CRP levels in RA patients from the Rheuma-VOR cohort divided by RF and ACPA pre- and absence. Statistical differences between groups were calculated using Mann-Whitney test. **(G)** Serum zonulin levels in healthy blood donors (BD, n=20), patients newly diagnosed with non-rheumatic disease (NRD, n=22), rheumatoid arthritis (RA, n=33), psoriatic arthritis (PsA, n=48), axial spondyloarthritis (axSpA, n=30), reactive arthritis (ReA, n=10), as well as IBD patients with active ulcerative colitis (UC A, n=14), active Crohn’s disease (CD A, n=13), and Crohn’s disease under remission (CD R, n=12). Dots in the Rheuma-VOR groups depict single measurements, while dots in the IBD groups represent median values calculated from 2 replicates. **(H)** Serum zonulin in Rheuma-VOR RA patients clustered by RF and ACPA positivity. Statistical differences between groups were calculated using Mann-Whitney test. Data is shown as mean ± SD, *p < 0.05; **p < 0.01; ***p < 0.001; ****p < 0.0001; not significant if not further indicated.

Calprotectin levels were determined in all samples using a commercially available ELISA kit. While the FDR 1 group served as control for the other groups from the SCREEN-RA/SCQM cohorts, we included the NRD serum samples as well as the BD sera as controls for the Rheuma-VOR patients. In line with previously published data, calprotectin levels were elevated in cRA patients (**Figure 1A**) and in new-onset RA patients (**Figure 1B**). However, we did neither detect a significant increase in calprotectin levels in first-degree individuals at risk for developing RA, nor in the different SpA subforms, when comparing to the respective control groups. This indicates calprotectin to be rather specifically increased in patients with new onset or chronic RA, but not in earlier disease phases. Moreover, in contrast to previous studies we were not able to detect elevated calprotectin levels in newly diagnosed SpA. To further compare calprotectin levels with the commonly used inflammatory marker C-reactive protein (CRP), we obtained serum CRP values of patients from the Rheuma-VOR cohort. Serum CRP levels were significantly increased in RA and PsA patients compared to the NRD control group (**Figure 1C**) and positively correlated with serum calprotectin concentration (**Figure 1D**). As RF and ACPA autoantibodies have been associated to disease activity and inflammation in RA patients (Aletaha et al., 2015; de Brito Rocha et al., 2019), we divided FDR individuals and cRA patients from the SCREEN-RA/SCQM cohort as well as new-onset RA patient from the Rheuma-VOR cohort based on their RF and ACPA seropositivity and investigated potential differences in serum calprotectin and CRP levels. ACPA and RF presence both resulted in higher calprotectin concentrations in individuals from the SCREEN-RA/SCQM cohort, although differences between RF positive and negative individuals did not reach significance (**Figure 1E**). In contrast, calprotectin levels in new onset RA patients from the Rheuma-VOR cohort did not differ based on RF and ACPA presence/absence. Similarly, serum CRP levels did not differentiate RF or ACPA seropositive and –negative RA patients (**Figure 1F**). These results however need to be interpreted with caution, as the division of RA patients from the Rheuma-VOR cohort based on autoantibody presence resulted in a low sample size in the RF and ACPA negative group. Overall, this data suggests seropositivity in ACPA to be associated to systemic inflammation measured by calprotectin.

With the aim to evaluate zonulin levels, a marker for intestinal permeability, in new-onset RA patients and to compare them to patients with other newly-diagnosed rheumatic diseases, zonulin levels were quantified in 163 serum samples from the Rheuma-VOR cohort using a commercial ELISA kit. The samples from the SCREEN-RA/SCQM cohorts were not suitable for this analysis due to their extended storage (>3 years) at -80°C, which interferes with the quantification of zonulin. Notably, we detected significantly increased zonulin concentrations in the serum of new-onset RA patients compared to blood donors (**Figure 1G**). Interestingly, the zonulin levels of PsA, axSpA, ReA, and NRD patients were slightly increased compared to the BD controls, but did not differ significantly. In order to compare zonulin levels in these cohorts to patients with a disease affecting the gastrointestinal mucosa, serum samples from IBD patients divided into active ulcerative colitis (UC A, n=14), active Crohn’s disease (CD A, n=13) and Crohn’s disease in remission (CD R, n=12) were further included as controls (metadata in **Table 1**). While zonulin secretion in the rheumatic groups were similar to active IBD patients, CD patients in remission overall displayed the highest levels. Hence, we could corroborate previous findings identifying increased serum zonulin levels already in new-onset RA patients, but determined that they are not elevated in other rheumatic diseases. That strengthens zonulin as another potential specific biomarker for RA and suggests that RA and SpA differ in the presence of biomarkers reflecting acute mucosal inflammation during the new-onset phase before the initiation of treatment. Investigation of serum zonulin levels in RF and ACPA seropositive and seronegative patients revealed significantly increased zonulin levels in RF positive patients and a minor increase in RA patients with ACPA autoantibodies (**Figure 1H**). This indicates intestinal permeability to correlate with RA autoantibody seropositivity.

### 3.2 Serum IgG and IgA responses against oral pathobionts and several *P. copri* isolates

**Figure 2:**
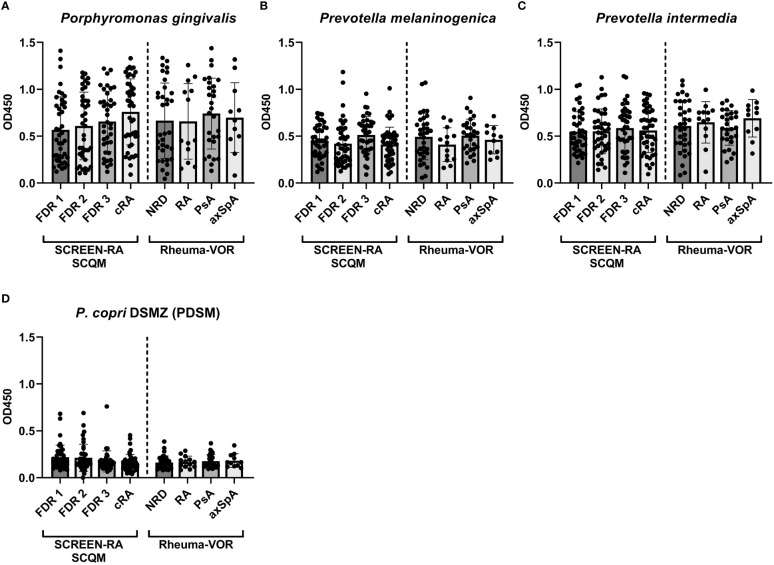
Serum IgG responses against oral pathobionts and the *P. copri* type strain in individuals at risk of RA, new onset rheumatic patients and patients with chronic RA. **(A-D)** IgG responses against the oral bacterial species *Porphyromonas gingivalis* **(A)**, *Prevotella melaninogenica* **(B)**, *Prevotella intermedia* **(C)** and against the *P. copri* type strain (PDSM) **(D)** in the serum of seronegative, symptom-free first-degree relatives (FDR 1, n=42), individuals with RA specific autoantibodies (FDR 2, n=40), individuals with RA specific symptoms (FDR 3, n=38) and chronic RA patients (cRA, n=45) as well as in patients with non-rheumatic disease (NRD, n=34) or new onset of rheumatoid arthritis (RA, n=13), psoriatic arthritis (PsA, n=28), and axial spondyloarthritis (axSpA, n=11). Data is shown as mean ± SD, differences are not statistically significant if not further indicated.

In order to extend the characterization of biomarkers to differentiate rheumatic diseases in our cohort, an ELISA assay was established to determine the serum IgG reactivity against previously described oral and gut-derived bacteria. To this end, the IgG antibody responses against the four bacterial strains *Prevotella copri, Porphyromonas gingivalis, Prevotella intermedia, and Prevotella melaninogenica* were measured in serum samples from the SCREEN-RA/SCQM studies as well as a subset of serum samples from the Rheuma-VOR cohort (**Table 2**). In contrast to previous studies, we did neither detect elevated IgG responses in cRA or NORA patients against the three oral pathobionts *P. gingivalis* (**Figure 2A**), *P. melaninogenica* (**Figure 2B**), and *P. intermedia* (**Figure 2C**), nor against the *P. copri* type strain (further named PDSM) (**Figure 2D**), when being compared to other patients or respective control groups. Moreover, none of the other preclinical or new onset patient groups showed significant differences in their IgG response compared to controls.

**Table 2:** Serum samples used in anti-bacterial ELISA.

Cohort	SCREEN-RA/SCQM				Rheuma-VOR			
patient group	FDR 1	FDR 2	FDR 3	cRA	NRD	RA	PsA	axSpA
**total numbers, n (female %)**	42 (76)	40 (88)	38 (90)	45 (78)	34 (56)	13 (69)	28 (50)	11 (64)
**age, median**	54	54	59	56	46	51	60	33
**ACPA positivity, n**	0	25	9	31	0	7	0	0
**RF positivity, n**	0	19	8	34	1	6	0	0

**Figure 3:**
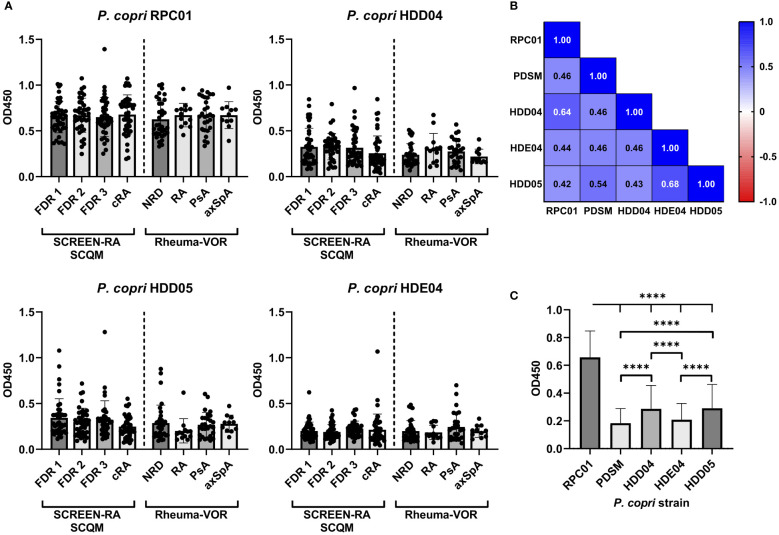
Serum IgG responses against distinct, genetically different *P. copri* strains in individuals at risk of RA, new onset rheumatic patients and patients with chronic RA. **(A)** IgG responses against the different *P. copri* strains RPC01, HDD04, HDE04, and HDD05 in serum samples of seronegative, symptom-free first degree relatives (FDR 1, n=42), individuals with RA specific autoantibodies (FDR 2, n=40), individuals with RA specific symptoms (FDR 3, n=38) and chronic RA patients (cRA, n=45) as well as in patients with non-rheumatic disease (NRD, n=34) or new onset of rheumatoid arthritis (RA, n=13), psoriatic arthritis (PsA, n=28), and axial spondyloarthritis (axSpA, n=11). **(B)** Correlation matrix showing Spearman’s correlation coefficient (r) of serum IgG responses against the *P. copri* strains RPC01, PDSM, HDD04, HDE04, and HDD05, P value of all comparisons < 0.0001 (****). **(C)** Averaged serum IgG responses against *P. copri* strains, independent of disease grouping, data analyzed using Friedman test. Data indicates mean ± SD, ****p < 0.0001, not significant if not further indicated.

Since previous studies investigating the antibody reactivity of *P. copri* solely focused on the type strain isolated from a healthy Japanese donor (Hayashi et al., 2007), we expanded the *P. copri* strain panel to compare the immunostimulatory potential of additional distinct strains belonging to the *P. copri* complex. Specifically, we tested the serum antibody binding capacity of four additional *P. copri* strains from our internal laboratory collection, which were previously isolated from fecal samples of healthy individuals (HDD04, HDE04 and HDD05) and from a patient with new onset of rheumatoid arthritis (RPC01). While the strains *P. copri* PDSM, HDD04 and RPC01 clustered based on the previous defined classifications (Tett et al., 2019) into clade A, strain HDE04 and HDD05 belonged to clade C and D, respectively. Whole genome phylogeny of the five strains within the *P. copri* complex are depicted in the **supplementary material** (**Supplementary Figure 1**). Similar to our results with strain PDSM, the IgG reactivity against RPC01, HDD04, HDE04 and HDD05 did not differ between distinct rheumatic groups, neither between patient groups from the SCREEN-RA/SCQM nor from the Rheuma-VOR cohort (**Figure 3A**). However, using Spearman’s correlation analysis, a highly significant (P < 0.0001) moderate to strong positive correlation (r = 0.42 – 0.64) was detected between the five *P. copri* strains (**Figure 3B**). Remarkably, by comparing the overall strain-specific IgG antibody reactivity in all serum samples, we found highly significant differences between all the *P. copri* isolates, with *P. copri* strain RPC01 clearly reaching the highest signals (**Figure 3C**). We obtained similar results measuring IgA reactivity profiles against all five *P. copri* strains (**Supplementary Figure 2**). While IgA responses did not significantly differ between different disease types from the SCREEN-RA/SCQM or the Rheuma-VOR cohort (**Supplementary Figures 2A, B**), we also identified the highest IgA signal in both cohorts, independently of disease status, against the *P. copri* strain RPC01 (**Supplementary Figures 2C, D**). Together, this demonstrates a high variability between *P. copri* strains in their ability to trigger immune responses in their host, which appears not to be driven by subspecies clustering. Moreover, the observed increased immune recognition of *P. copri* RPC01 compared to other strains suggests a higher immunogenic potential of the RA patient strain compared to strains isolated from healthy individuals.

### 3.3 Potential factors modulating serum IgG responses against *P. copri* strains

To further understand whether current *P. copri* colonization and abundance levels influence anti-*P. copri* IgG antibody levels, we analyzed the abundance of *P. copri* in the fecal samples of the respective patients from the Rheuma-VOR and SCQM study by 16S rRNA gene sequencing and included previously published sequencing data from the SCREEN-RA study (Alpizar-Rodriguez et al., 2019).

**Figure 4:**
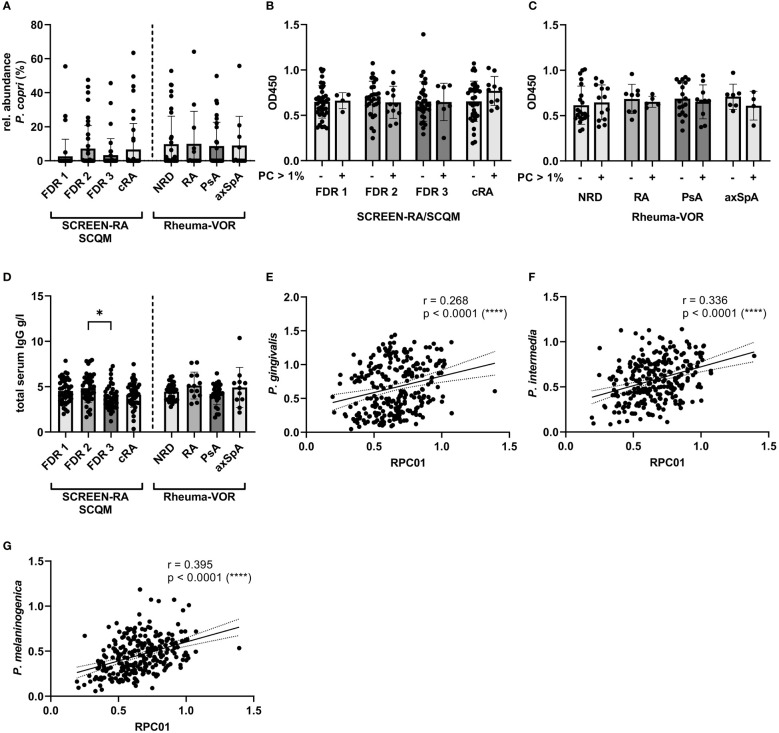
Fecal *P. copri* abundances and total serum IgG concentration in individuals at risk of RA, new onset rheumatic patients and patients with chronic RA. **(A)** Relative abundances of *Prevotella copri* in fecal samples of participants from the SCREEN-RA/SCQM and Rheuma-VOR cohorts determined by 16S rRNA gene sequencing. B-C: Serum IgG responses against *P. copri* RPC01 divided by presence (rel. abundance > 1%) and absence (rel. abundance < 1%) of *P. copri* in fecal samples of the respective patients from the SCREEN-RA/SCQM **(B)** and Rheuma-VOR **(C)** cohorts. **(D)** Total serum IgG concentration of patients from the SCREEN-RA/SCQM and Rheuma-VOR cohorts. **(E–G)** Spearman correlation between serum IgG responses against RPC01 and the oral pathobionts *P. gingivalis* **(E)**, *P. intermedia* **(F)**, and *P. melaninogenica* **(G)**. Samples from SCREEN-RA/SCQM: FDR 1, n=42; FDR 2, n=40; FDR 3, n=38; cRA, n=45). Samples from Rheuma-VOR cohort: NRD, n=34; RA, n=13; PsA, n=28; axSpA, n=11. Data indicates mean ± SD, *p < 0.05; ****p < 0.0001, not significant if not further indicated.

Hypothesizing that a higher abundance or general presence of *P. copri* in the intestine of patients is able to trigger the immune system and eventually provokes the production of *P. copri* specific IgG in the serum of patients, we expected the serum antibody responses to correlate with intestinal prevalence. However, the relative abundance of *P. copri* in feces within our sample collections did neither significantly differ between FDR 1, FDR 2, FDR 3 or cRA patients, nor between new-onset patients with NRD, RA, PsA or axSpA (**Figure 4A**). Correlation between serum IgG response against *P. copri* strain RPC01 (chosen as representative of the five *P. copri* strains) and the relative abundance of fecal *P. copri* did not detect a significant relation between both variables in the different patient groups from SCREEN-RA/SCQM or Rheuma-VOR (Spearman’s correlation p value not significant, **Table 3**). Moreover, presence (relative abundance > 1%) or absence of *P. copri* in the fecal samples did not have an effect on the serum IgG responses against RPC01 in the different patient groups (**Figures 4B, C**). Hence, the current colonization of *P. copri* in the intestine unexpectedly appeared to not influence its serological recognition by the immune system.

**Table 3:** Spearman correlation between IgG response against *P. copri* strain RPC01 and relative abundance of fecal *P. copri* or total serum IgG.

Cohort	All samples	SCREEN-RA/SCQM				Rheuma-VOR			
Patient group		FDR 1	FDR 2	FDR 3	cRA	NRD	RA	PsA	axSpA
***P. copri*** **rel. abundance**	r = -0.023	r = 0.216	r = -0.235	r = 0.005	r = 0.196	r = 0.066	r = -0.414	r = 0.037	r = -0.195
	P = 0.709 (ns)	P = 0.17 (ns)	P = 0.144 (ns)	P = 0.977 (ns)	P = 0.53 (ns)	P = 0.712 (ns)	P = 0.161 (ns)	P = 0.853 (ns)	P = 0.561 (ns)
**total serum IgG**	r = 0.159	r = 0.078	r = 0.054	r = 0.268	r = 0.382	r = -0.021	r = -0.28	r = 0.078	r = 0.20
	P = 0.011 (*)	P = 0.625 (ns)	P = 0.745 (ns)	P = 0.104 (ns)	P = 0.009 (**)	P = 0.907 (ns)	P = 0.354 (ns)	P = 0.696 (ns)	P = 0.557 (ns)

In order to evaluate whether anti-*P. copri* IgG responses merely reflected total IgG levels in individuals, total IgG levels were determined in the sera of study participants. The comparison of total serum IgG levels between patient groups revealed a slight difference between the mean IgG concentrations in the serum of FDR 2 and FDR 3 individuals, but other diseases and disease subgroups did not differ from each other (**Figure 4D**). Importantly, linking the RPC01-specific IgG responses with the serum IgG concentrations for all participants of the study did identify a weak positive correlation between these two variables (Spearman correlation r = 0.159, P = 0.011 (*), **Table 3**). However, when dividing samples based on diagnoses, we only observed a significant, moderate positive correlation between both variables in the cRA group (Spearman r = 0.382, P = 0.009 (**)), but not others. We furthermore analyzed whether antibody levels correlated between different species of bacteria, which could reflect a generalized immune-reactivity against bacteria in individuals. We observed highly significant, weak to moderate positive correlations between serum IgG responses against RPC01 and *P. gingivalis* (**Figure 4E**), *P. intermedia* (**Figure 4F**), and *P. melaninogenica* (**Figure 4G**) across all samples, which was also partially reflected in the individual patient groups (**Table 4**). In summary, these results suggest that serum IgG levels marginally determine the reactivity against *P. copri* across the different patient groups and that serum immune reactivity correlates between distinct bacterial species colonizing different mucosal sites.

**Table 4:** Spearman correlation between IgG response against *P. copri* strain RPC01 and IgG response against oral pathobionts.

Cohort	All samples	SCREEN-RA/SCQM				Rheuma-VOR			
Patient group		FDR 1	FDR 2	FDR 3	cRA	NRD	RA	PsA	axSpA
**IgG response** ***P. gingivalis***	r = 0.268	r = 0.223	r = 0.220	r = 0.041	r = 0.376	r = 0.444	r = 0.181	r = 0.417	r = -0.027
	P < 0.0001 (****)	P = 0.155 (ns)	P = 0.13 (ns)	P = 0.808 (ns)	P = 0.011 (*)	P < 0.01 (**)	P = 0.554 (ns)	P = 0.027 (*)	P = 0.946 (ns)
**IgG response** ***P. intermedia***	r = 0.337	r = 0.371	r = 0.364	r = 0.073	r = 0.496	r = 0.512	r = 0.275	r = 0.194	r = 0.200
	P < 0.0001 (****)	P = 0.016 (*)	P = 0.021 (*)	P = 0.665 (ns)	P < 0.001 (***)	P < 0.01 (**)	P = 0.363 (ns)	P = 0.321 (ns)	P = 0.557 (ns)
**IgG response** ***P. melaninogenica***	r = 0.395	r = 0.323	r = 0.574	r = 0.252	r = 0.439	r = 0.571	r = 0.374	r = 0.432	r = 0.127
	P < 0.0001 (****)	P = 0.037 (*)	P < 0.001 (***)	P = 0.127 (ns)	P < 0.01 (**)	P < 0.001 (***)	P = 0.21 (ns)	P = 0.022 (*)	P = 0.714 (ns)

**Figure 5:**
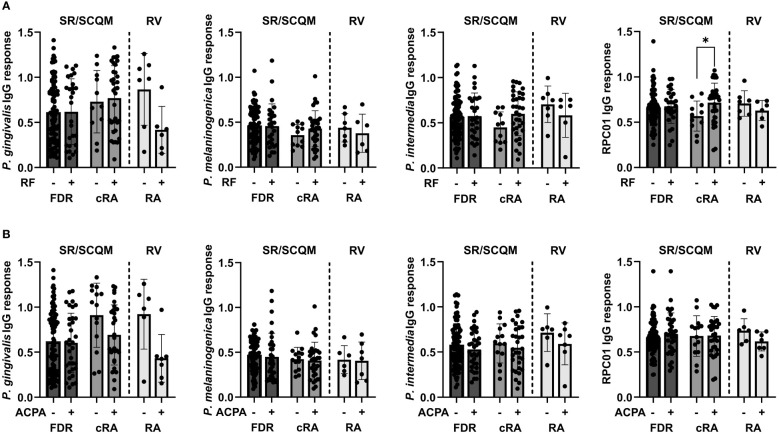
Serum IgG responses against oral pathobionts and *P. copri* based on RA-specific autoantibody seropositivity. **(A, B)** IgG responses against *P. gingivalis*, *P. melaninogenica*, *P. intermedia* and *P. copri* RPC01 in FDR and cRA patients from SCREEN-RA/SCQM cohort and new onset RA patients from the Rheuma-VOR cohort divided by RF **(A)** and ACPA **(B)** autoantibody seropositivity. Statistical differences between positive and negative groups within patient groups were calculated using Mann-Whitney test. Data indicates mean ± SD, *p < 0.05, not significant if not further indicated.

Lastly, we assessed the potential influence of autoantibody presence or absence on the antibacterial IgG responses in the serum of FDR and cRA patients from SCREEN-RA/SCQM and new onset RA patients from Rheuma-VOR. Seropositivity for RF did not affect antibody reactivity against the oral pathobionts *P. gingivalis*, *P. melaninogenica* and *P. intermedia* but strikingly increased the response against *P. copri* strain RPC01 in chronic RA patients (**Figure 5A**). This effect was specific for RF, as we did not detect any differences in the IgG responses against the four bacteria based on ACPA seropositivity (**Figure 5B**). Hence, this data suggests the presence of RF autoantibodies in RA patients to enhance serological detection of *P. copri* and possibly indicates towards a direct interaction between RF autoantibodies and *P. copri* cells.

## 4 Discussion

In the present study, we investigated serum levels of calprotectin and zonulin, two biomarkers associated with systemic and intestinal inflammation in rheumatic diseases, in individuals at risk for developing RA, chronic RA patients and patients with new onset of RA or SpA. We could confirm previous results (Nielsen et al., 2018; Jarlborg et al., 2020) by detecting an increased calprotectin titer in chronic RA patients. While higher calprotectin levels in the serum of chronic axSpA and PsA patients have been previously reported in literature (Jarlborg et al., 2020), we did not observe an increase in these disease groups in our cohorts. That suggested that systemic inflammation during disease onset in SpA subforms is reduced compared to chronic SpA and is not as advanced as in NORA. Consistent with previous findings (Tajik et al., 2020), we also found serum zonulin to be specifically enriched in NORA patients. Due to instability of zonulin in serum after long term storage of samples, we could not assess zonulin levels in the serum of individuals from the SCREEN-RA/SCQM cohort, which clearly would have been valuable and should be followed up in the future. Nevertheless, in line with previously generated data our results identified serum calprotectin as well as zonulin as interesting biomarkers in rheumatic disorder diagnostic. It needs to be noted here, that the assessment of intestinal permeability by measuring serum zonulin in commercial ELISA kits has recently been questioned due to the detection of unspecific binding in addition to the target protein zonulin/prehaptoglobin-2 (Ajamian et al., 2019). However, level of calprotectin and zonulin respectively correlated with ACPA and RF seropositivity in pre- and chronic RA patients, which substantiates previous studies linking RA specific autoantibodies to enhanced inflammation (Aletaha et al., 2015; ten Brinck et al., 2018).

When we further investigated serum IgG detection of the oral bacteria *P. gingivalis*, *P. intermedia*, and *P. melaninogenica* as well as the *P. copri* type strain (PDSM) within samples from our cohorts, we were unable to reproduce previous data showing an increased antibody response against these bacteria in RA patients (Ogrendik et al., 2005; Pianta et al., 2017). However, our results agree with another study (Manoil et al., 2021) failing to detect IgG reactivity against *P. gingivalis* and *P. intermedia* in preclinical RA stages. It should be noted, that these previously conducted studies did neither consider microbial compositions nor abundances of the assayed bacteria in the oral cavity or the intestine. It can be assumed that microbial interaction with the immune system is at least partially determined by the prior presence or absence of these specific bacteria in microbial communities. Differences in the bacterial composition between participants from our and the above-mentioned cohorts are highly plausible due to the fact that these studies were conducted with participants coming from different sites of the world. Geographic origin of individuals is known to have a significant effect on the gut microbial composition, as it has been shown when comparing the microbial composition of human cohorts from different countries and continents (Yatsunenko et al., 2012; Rehman et al., 2016), and even from different regions within one single country (Sun et al., 2020). Comparing our results from German and Swiss cohorts with data acquired from the US (Pianta et al., 2017) or Australia and Turkey (Ogrendik et al., 2005) is therefore highly interesting but also suffers from confounding factors. Hence, microbial composition is an important factor to explain the lack of reproducibility between different studies, and should be carefully taken into account during future study conduct and further data interpretation.

Additionally to investigating serum antibody responses against the *P. copri* type strain (PDSM), we expanded the assay using four distinct *P. copri* strains from our internal lab collection. We detected differences in the immunostimulatory potential between the isolates and strikingly the highest immunoglobulin reactivity against *P. copri* strain RPC01, a strain that was isolated from a new onset RA patient. That suggested strain RPC01 to be more capable of activating the immune system, potentially induced by pathogenic genes integrated within its genome. To investigate that hypothesis further and to evaluate whether *P. copri* strains from RA patients generally differ from *P. copri* strains present in healthy individuals, isolation of additional strains combined with thorough genetic characterization by whole genome sequencing analysis should be performed. Moreover, evaluating the effect of genetically different *P. copri* strains as well as isolates from RA patients and healthy individuals on the host by *in vivo* experiments would provide additional insights. While our data in line with previous studies depicts that *P. copri* is recognized by serum immunoglobulins, further identification of antigens responsible for the immune reaction would be substantially important and should be subject of subsequent studies. Interestingly, we identified a specifically increased immune stimulation by *P. copri* in RF positive pre- and chronic RA patients. This corroborates the hypothesis that specific bacteria can cross-react with autoantibodies in RA enhancing the immune reaction and driving inflammation (Derksen et al., 2017) and particularly sheds light on the role of *P. copri* as autoantigen.

Notably, the design of this study has the following limitations. Firstly, although sample sizes of the SCREEN-RA/SCQM cohort facilitated to study bacterial immune modulation precisely in different stages of RA development, patient groups from the Rheuma-VOR cohort were in contrast mostly limited and unequal in numbers. Therefore, antibody responses against *P. copri* and oral pathobionts in disease onset of the different rheumatic diseases need to be interpreted with caution and interactions between bacteria and the immune system in these rheumatic subgroups require further investigations in the future. Secondly, the here applied method for detecting the immunoreactive potential of intestinal and oral microbes solely focused on surface antigens of the bacterium without being able to detect immune stimulation by non-surface antigens e.g. secretion products. Additionally, this assay only enabled to investigate immune stimulation by bacteria grown *in vitro*, whereas their antigen repertoire *in vivo* in a complex microbial environment presumably differs enormously. Lastly, knowing about the vast diversity and heterogeneity within the *P. copri* species complex (Tett et al., 2019), the here investigated immune reaction to *P. copri* is still largely limited and explicitly necessitates greater consideration in the future.

Nonetheless, our study expanded a previously established *P. copri* antibody-binding assay by testing additional, distinct strains including a strain from an RA patient. This revealed heterogeneous strain specific effects in the immune modulation capacity within the species, which will have to be taken into account when further evaluating the role of *P. copri* in health and disease. However, we were not able to reproduce several previous findings identifying anti-*Prevotella* immunity during early disease stages. Thus, our data does not support the hypothesis of a systemic adaptive immune reaction against *P. copri*, or other *Prevotella* species, in the context of early or established RA.

## Data availability statement

The datasets presented in this study can be found in online repositories. The names of the repository/repositories and accession number(s) can be found in the article/**Supplementary Material**.

## Ethics statement

The studies involving human participants were reviewed and approved by Ethics commisions of the Hannover Medical School and the University Hospital Hamburg-Eppendorf. The patients/participants provided their written informed consent to participate in this study.

## Author contributions

LA, TW, AF and TS contributed to conception and design of the study. LA performed all experiments. BG, PP, MB, and SH contributed biosamples from clinical studies. LA performed the statistical analysis. LA wrote the first draft of the manuscript. All authors contributed to manuscript revision, read, and approved the submitted version.

## Supplementary material

Supplementary Figure 1 Phylogeny of *P. copri* strains used in this study. Phylogenetic neighbor-joining tree of *P. copri* isolates used in this study (P. copri DSM, RPC01, HDD04, HDE04, HDD05, marked in blue) as well as reference strains reported by Tett et al., 2019. Numbers at the nodes represent the respective bootstrap probability.

Supplementary Figure 2 Serum IgA responses against distinct, genetically different *P. copri* strains in individuals at risk for RA, new onset rheumatic patients and patients with chronic RA. **(A, B)** IgA responses against the *P. copri* strains PDSM, RPC01, HDD04, HDE04, and HDD05 in **(A)** serum samples of seronegative, symptom-free first-degree relatives (FDR 1, n=42), individuals with RA specific autoantibodies (FDR 2, n=40), individuals with RA specific symptoms (FDR 3, n=38) and chronic RA patients (cRA, n=45) as well as in **(B)** serum samples from patients with non-rheumatic disease (NRD, n=34) or new onset of rheumatoid arthritis (RA, n=13), psoriatic arthritis (PsA, n=28), and axial spondyloarthritis (axSpA, n=11). **(C, D)** Averaged IgA responses against *P. copri* strains in the serum of **(C)** SCREEN-RA/SCQM and **(D)** Rheuma-VOR, independent of disease grouping, data analyzed using Friedman test. Data indicates mean ± SD, *: p<0.05; **: p<0.01; ***: p<0.001; ****: p<0.0001, not significant if not further indicated.
