# Supplementary figures and images for: Characterization of serum biomarkers and antibody responses against Prevotella spp. in preclinical and new-onset phase of rheumatic diseases
Source: Front Cell Infect Microbiol. 2023 Jan 18;12:1096211. doi: 10.3389/fcimb.2022.1096211 (PMC9889664; doi:10.3389/fcimb.2022.1096211)

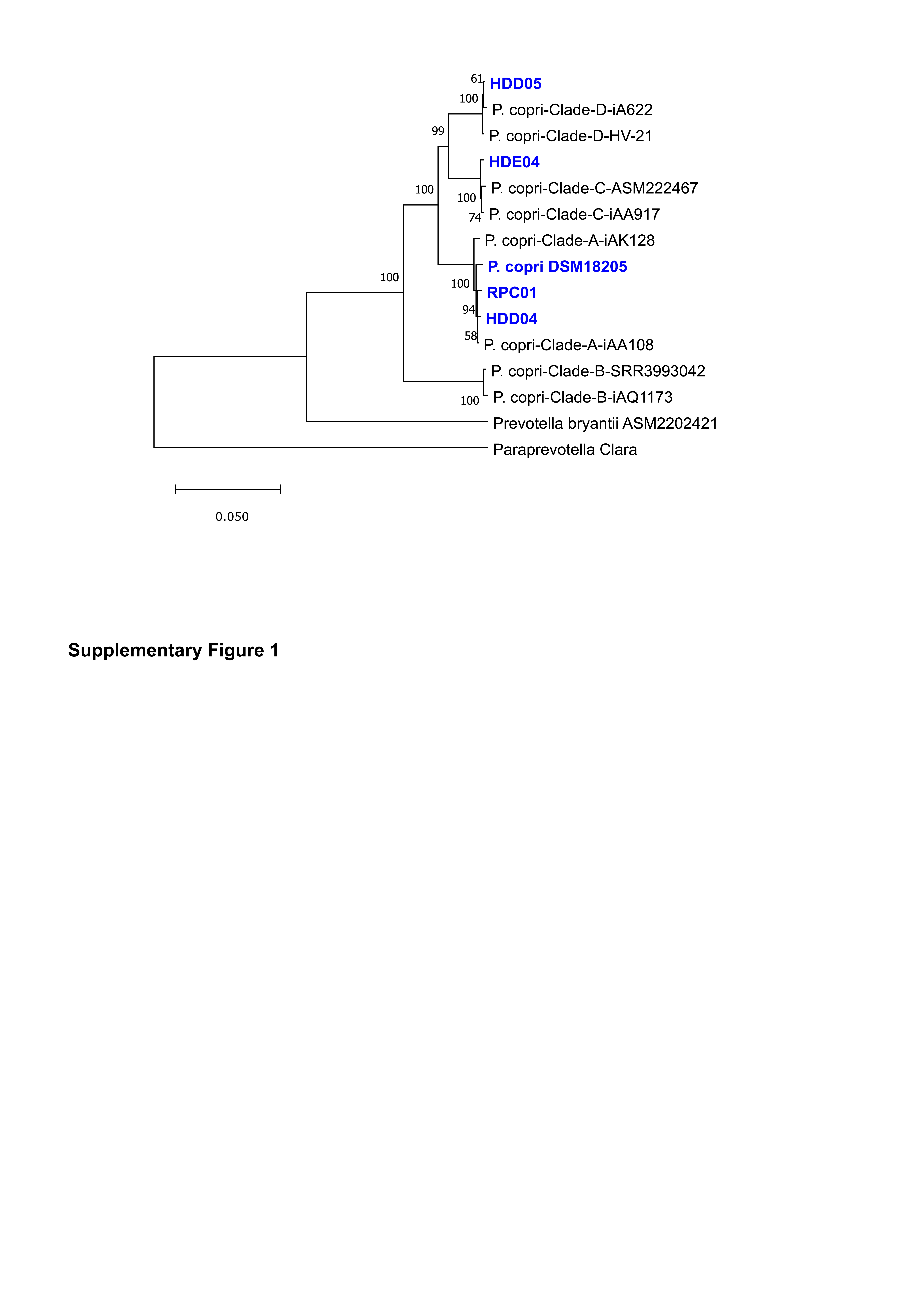

Supplement: Supplementary Figure 1 — Phylogeny of P. copri strains used in this study. Phylogenetic neighbor-joining tree of P. copri isolates used in this study (P. copri DSM, RPC01, HDD04, HDE04, HDD05, marked in blue) as well as reference strains reported by Tett et al., 2019. Numbers at the nodes represent the respective bootstrap probability. [file Image_1.jpg]

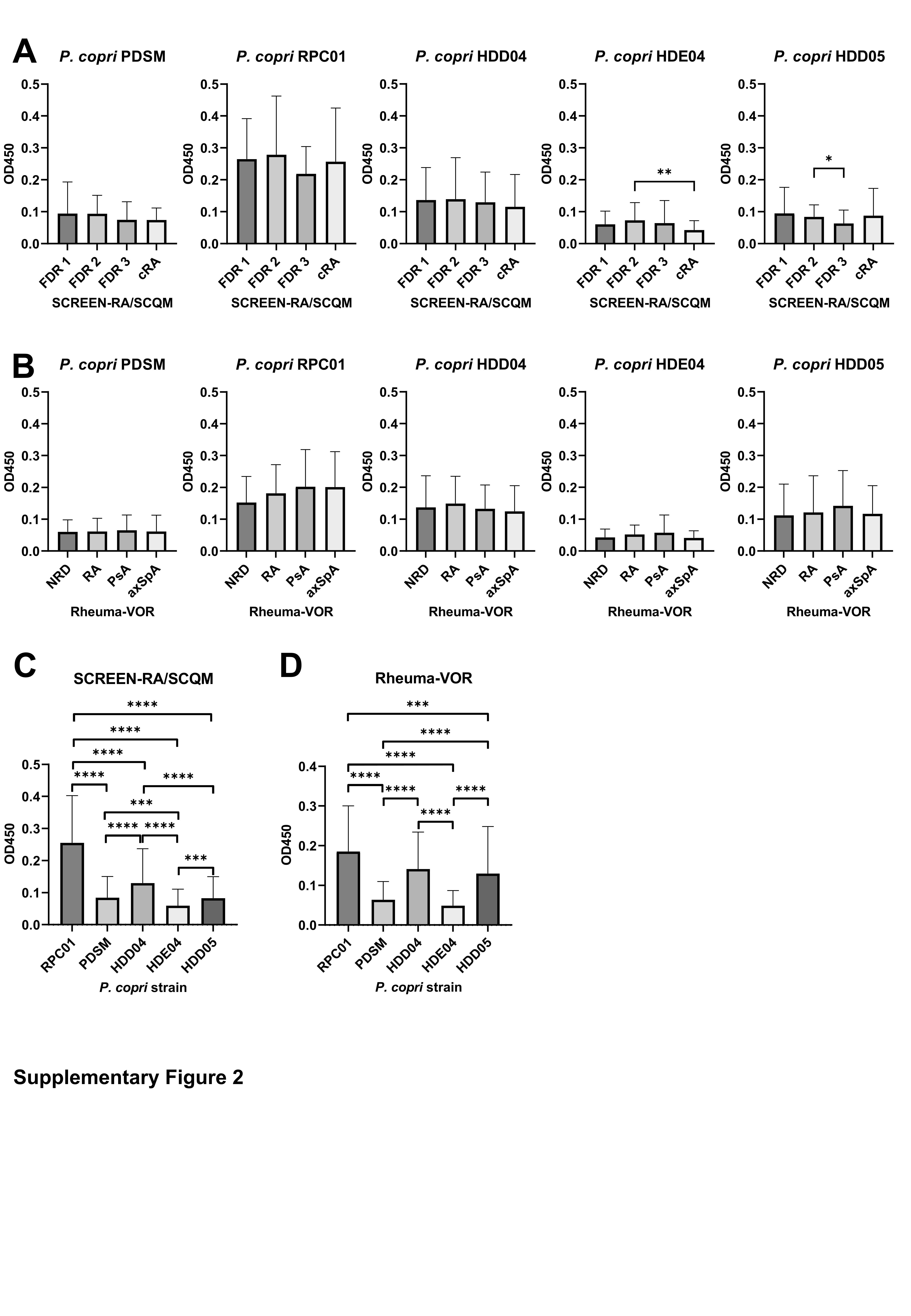

Supplement: Supplementary Figure 2 — Serum IgA responses against distinct, genetically different P. copri strains in individuals at risk for RA, new onset rheumatic patients and patients with chronic RA. (A, B) IgA responses against the P. copri strains PDSM, RPC01, HDD04, HDE04, and HDD05 in (A) serum samples of seronegative, symptom-free first-degree relatives (FDR 1, n=42), individuals with RA specific autoantibodies (FDR 2, n=40), individuals with RA specific symptoms (FDR 3, n=38) and chronic RA patients (cRA, n=45) as well as in (B) serum samples from patients with non-rheumatic disease (NRD, n=34) or new onset of rheumatoid arthritis (RA, n=13), psoriatic arthritis (PsA, n=28), and axial spondyloarthritis (axSpA, n=11). (C, D) Averaged IgA responses against P. copri strains in the serum of (C) SCREEN-RA/SCQM and (D) Rheuma-VOR, independent of disease grouping, data analyzed using Friedman test. Data indicates mean ± SD, *: p<0.05; **: p<0.01; ***: p<0.001; ****: p<0.0001, not significant if not further indicated. [file Image_2.jpg]
